# *De novo* mutation in a male patient with Fabry disease: a case report

**DOI:** 10.1186/1756-0500-7-11

**Published:** 2014-01-07

**Authors:** Francesco Iemolo, Federica Pizzo, Giuseppe Albeggiani, Carmela Zizzo, Paolo Colomba, Simone Scalia, Caterina Bartolotta, Giovanni Duro

**Affiliations:** 1 CNR-IBIM: Institute of Biomedicine and Molecular Immunology “A. Monroy”, Via Ugo la Malfa n.153, Palermo, Italy; 2 Department of Neurology, “R. Guzzardi” Hospital, Via Papa Giovanni XXIII, Vittoria, Ragusa, Italy; 3 Dipartimento Biomedico di Medicina Interna e Specialistica, University of Palermo, Via del Vespro 141, Palermo, Italy

**Keywords:** Fabry disease, α-galactosidase A, GLA gene, D165H mutation, *De novo* mutation

## Abstract

**Background:**

Fabry disease is an X-linked inherited metabolic condition where the deficit of the α-galactosidase A enzyme, encoded by the GLA gene, leads to glycosphingolipid storage, mainly globotriaosylceramide. To date, more than 600 mutations have been identified in human GLA gene that are responsible for FD, including missense and nonsense mutations, small and large deletions. Such mutations are usually inherited, and cases of *de novo* onset occur rarely.

**Case presentation:**

In this article we report an interesting case of a 44-year-old male patient suffering from a severe form of Fabry disease, with negative family history. The patient showed signs such as cornea verticillata, angiokeratomas, cardiac and neurological manifestations, an end-stage renal disease and he had low α-galactosidase A activity. We detected, in this subject, the mutation c.493 G > C in the third exon of the GLA gene which causes the amino acid substitution D165H in the protein. This mutation affects the amino acid - belonging to the group of buried residues - involved, probably, in the preservation of the protein folding. Moreover, studies of multiple sequence alignment indicate that this amino acid is highly conserved, thus strengthening the hypothesis that it is a key amino acid to the enzyme functionality.

The study of the relatives of the patient showed that, surprisingly, none of the members of his family of origin had this genetic alteration, suggesting a *de novo* mutation. Only his 11-year-old daughter - showing acroparaesthesias and heat intolerance with reduced enzymatic activity - had the same mutation.

**Conclusions:**

We suggest that a non-inherited mutation of the α-galactosidase A gene is responsible for Fabry disease in the patient who had reduced enzyme activity and classical clinical manifestations of the disease. In a family, it is rare to find only one Fabry disease affected subject with a *de novo* mutation. These findings emphasize the importance of early diagnosis, genetic counselling, studying the genealogical tree of the patients and starting enzyme replacement therapy to prevent irreversible vital organ damage that occurs during the course of the disease.

## Background

Anderson-Fabry disease (OMIM 301500), or Fabry disease (FD), is an X-linked inherited metabolic condition characterized by a deficit in the activity of the α-galactosidase A enzyme, encoded by the GLA gene, which leads to glycosphingolipid storage, mainly globotriaosylceramide (Gb3) [1]. FD is one of the most important sphingolipidoses and it is considered a rare disease, with an incidence of 1 in 40,000, though it is probably more frequent than commonly believed [2,3]. The clinical manifestations are severer in hemizygous male subjects than in heterozygous female subjects, who are sometimes completely asymptomatic, in accordance with Lyon hypothesis of random X-chromosome inactivation [2,4]. To date, more than 600 mutations have been identified in human GLA gene that are responsible for FD, including missense and nonsense mutations, small and large deletions (Human Gene Mutation Database, http://www.hgmd.org). Such mutations are usually inherited and cases of *de novo* onset, i.e. arisen spontaneously, occur rarely [5].

The neutral glycosphingolipid storage in the lysosomes of cells within many tissues is responsible for the systemic nature of the disease. Clinical manifestations of Fabry disease start in childhood, and life-threatening renal, cardiac and cerebrovascular complications typically arise in adulthood [6,7]. Selective damage of epithelial tubular cells and glomerular epithelial cells induces chronic renal disease, with progression to end-stage renal disease (ESRD) as the patient gets older. ESRD generally appears during the third/fourth decade of life in hemizygous males. Fabry patients who developed ESRD can be treated with peritoneal dialysis or haemodialysis, and kidney transplantation from living and cadaver donors [8]. Enzyme replacement therapy (ERT) provides beneficial effects, by reducing Gb3 storage and preventing its further accumulation [8,9]. ERT is the current therapy, available for Fabry patients, and it consists of the intravenous infusion of synthetic enzyme. In most cases, it blocks the progression of the disease but it is not able to treat some symptoms. So it is important to diagnose early in the patient the disease and start the therapy, in order to prevent the most harmful consequences of the disease [10,11].

We report the case of a 44-year-old man affected by Fabry disease. He suffered from a severe clinical form of the disease, at the age of 40 he developed ESRD and he underwent kidney transplant at 42. We detected a *de novo* mutation in the codon 165 of the GLA gene of the patient, but neither his mother nor his mother’s relatives had this mutation. His daughter harboured also the same mutation and showed the clinical manifestations of the disease typical of childhood [12]. The affected subjects underwent both enzyme replacement therapy.

## Case presentation

A 44-year-old man with systemic manifestations came to our neurology unit because of a stroke. The analysis of his medical history revealed that, during childhood, he showed high rate of antistreptolysin O (ASO) titer, and he frequently suffered from acroparaesthesias, hypohidrosis, joint pains, cold and heat intolerance and attacks of fever.

At 18, whorled opacities in the cornea (cornea verticillata) were first observed.

At 19, the early manifestations of periumbilical and genital angiokeratomas appeared.

At 37, clinical and laboratory tests confirmed the presence of acute articular rheumatism. At the same age, he manifested episodes of vertigo, tinnitus, hearing impairment and sudden deafness which were believed to be caused by a neurinoma; however, this was not confirmed by magnetic resonance imaging (MRI).

At 40, hypertrophic cardiomyopathy and mitral valve insufficiency, proteinuria and a reduced creatinine clearance rate were observed. He underwent renal biopsy. Light microscopy examination of the renal biopsy revealed non-specific findings: segmental increases in mesangial cellularity and mesangial matrix were seen in 3/24 of the glomeruli. On physical examination, his blood pressure was 140/90 mmHg with a regular pulse rate.

**Figure 1:**
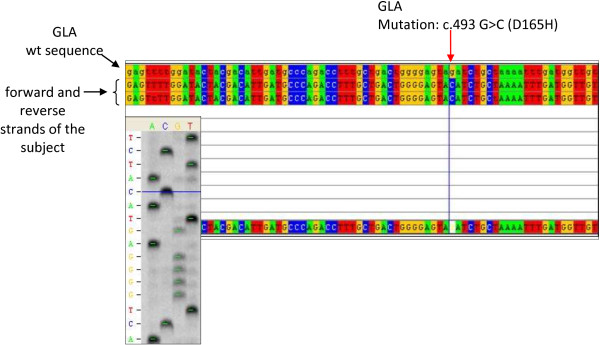
**Alignment of gene sequences.** Portion of the sequence of exon 3 of the GLA gene in Patient 2 (forward and reverse strands of the subject) aligned with the corresponding sequence of a healthy control (wt). The red arrow indicates the position of the mutation c.493 G > C (D165H in the protein). On the left, the sequencing gel image is shown.

**Figure 2:**
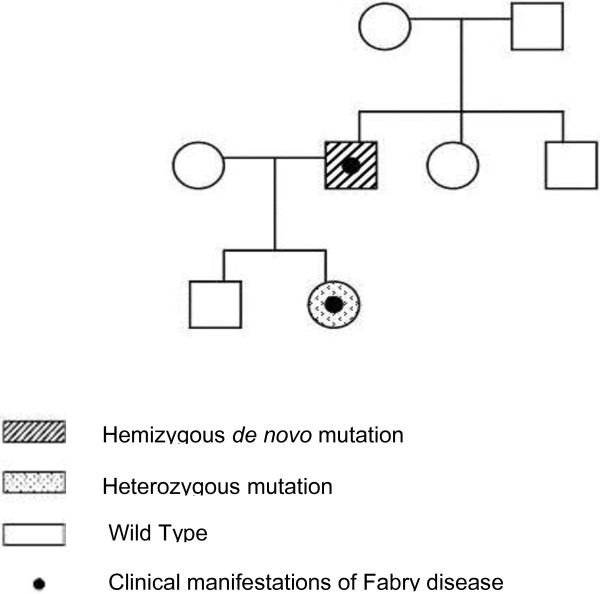
**Genealogical tree of the family of the patient.** The male patient (striped square) is the only member of its family of origin who had the mutation. In the next generation, his daughter harboured also the same mutation in heterozygosis (dotted circle).

From 37 to 42, the patient had four episodes of vertebrobasilar transient ischaemic attack. At 40, he underwent a complete diagnostic work-up, which revealed multiorgan involvement: damages in the CNS with silent ischemic strokes, both cortical and subcortical, and severe peripheral neuropathy; cardiac hypertrophy, altered repolarisation and right bundle branch block; regarding eyes, cornea verticillata was confirmed; renal involvement with kidney failure (stage II/DOQI CKD). Selective damage of epithelial cells and glomerular epithelial cells induced chronic renal disease with progression to ESRD. The same year, he started haemodialysis, and at 42 he underwent kidney transplant from a cadaver donor which normalised his kidney function. Because of all the clinical manifestations, the presence of Fabry disease was suspected and genetic and enzymatic analyses were performed. Peripheral blood sample was collected and DNA was extracted using the GenElute Blood Genomic DNA Kit (Sigma-Aldrich, USA). A pre-sequencing screening was performed on DNA sample by high resolution melting (HRM) analysis using the Light Cycler 480 system (Roche Applied Science, Germany) [13]. Purified PCR mutated products were sequenced using LI-COR NEN Model 4300 DNA Analyzer, according to the LI-COR protocol. Sequence analysis of the GLA gene in the patient, in comparison with the wild-type sequence (Figure 1), revealed a single nucleotide point mutation in hemizygosis at nucleotide c.493 G > C in exon 3 (D165H in the protein) [14]. The α-galactosidase A activity of the patient was determined by Dried Blood Filter Paper test described by Chamoles et al., with minor modifications [15]: enzyme activity in the whole blood was 0.5 nmol/h/ml (normal range is > 3.0 nmol/h/ml). We extended the genetic analysis to the family members of the patient (mother, sister and brother) (Figure 2) and none of them had any alteration in the GLA gene, suggesting a *de novo* mutation in the patient.

The 11-year-old daughter of the patient showed clinical manifestations referable to Fabry disease, too. She had severe acroparaesthesias, gastrointestinal symptoms, heat intolerance and the early signs of renal involvement (proteinuria). The results of the blood tests were in the normal range. Kidney B-mode ultrasound and color Doppler ultrasound failed to show significant discrepancies. Liver ultrasound was normal. The result of ophthalmological exams and the study of retinal funds were within the norm. Echocardiogram showed heart size, morphology, and function to be normal. Brain MRI was normal. Genetic and enzymatic studies were also performed on this subject: genetic analysis showed she had the same mutation of her father (c.493 G > C in exon 3 of the GLA gene) in heterozygosis and the α-galactosidase A activity was below the normal range (1 nmol/h/ml).

Both patients, the proband and his daughter, started ERT: the father had significant beneficial effects only on his acroparaesthesias and angiokeratomas; the daughter had marked clinical benefits on all manifestations thanks to the early diagnosis, according to literature [11].

## Discussion

Fabry disease is a rare pathology and significant therapeutic progress have been done in recent years. The heterogeneity of its manifestations causes a high degree of variation in its clinical symptoms, both in hemizygous and heterozygous.

In this paper we report the case of two members of one family - having the classic form of Fabry disease - in which the sequence analysis of the GLA gene, in comparison with the wild-type sequence, revealed a single nucleotide point mutation (hemizygous and heterozygous) at nucleotide c.493 G > C in exon 3. This mutation results in the replacement of an amino acid (aspartic acid) by a basic one (histidine) at codon 165, thus altering normal α-galactosidase A activity. This genetic alteration was previously described, in a Hungarian family, as associated with the classic form of Fabry disease [14].

**Figure 3:**
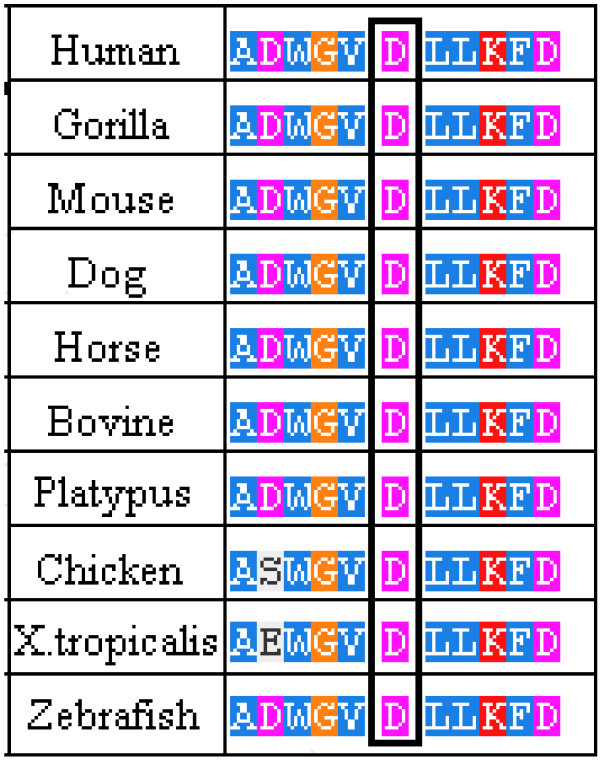
**Phylogenetic conservation of wild-type human α-Gal A amino acids.** Alignment of wild-type human α-Gal A amino acids with other organisms. The rectangle indicates the position of the amino acid that was found mutated in the patient.

The D165 amino acid belongs to the group of buried amino acids, so probably it is involved in the preservation of the protein folding, as reported in literature [14,16]. Moreover, we believe that this amino acid and the flanking ones have a key role in the enzyme functionality, as revealed also by the multiple sequence alignment, which shows that they have been highly conserved throughout the evolution (Figure 3). Our hypothesis is strengthened by the fact that the mutations affecting this amino acid (D165H e D165V) and some of the flanking ones (G163V, L166G e L166V) are responsible for the classic phenotype of Fabry disease [14,17-20]. Moreover, the study of the potential role of this mutation, performed using PolyPhen-2 software, confirms the damaging effect on the enzyme structure.

The study of the relatives of the patient showed, surprisingly, that none of his family members (mother, sister or brother) was affected by the disease; all of them showed normal enzymatic activity and the wild-type GLA gene. This indicated that the disease was caused by a *de novo* mutation, arisen spontaneously in this male patient. To confirm these findings, the analysis of the genetic profile of the patient and his relatives was carried out, proving that they had a family relationship. In a family, it is rare to find only one Fabry disease affected subject with a *de novo* mutation [21].

## Conclusion

In conclusion, we suggest that a non-inherited mutation of the α-galactosidase A gene is responsible for Fabry disease in the patient, who had reduced enzyme activity and classical signs of the disease, such as typical skin lesions, corneal opacities, and cardiac, renal and neurological manifestations. These findings emphasize the importance of early diagnosis, genetic counselling, studying the genealogical tree of the patients and starting the enzyme replacement therapy to prevent irreversible vital organ damage that occurs during the course of the disease.

## Consent

Written informed consent was obtained from the patients for the publication of these data and any accompanying images. A copy of the written consent is available for review by the Series Editor of this journal.

The study was approved by the University Hospital Ethics Committee.

## Abbreviations

FD: Fabry disease; Gb3: Globotriaosylceramide; ESRD: End-stage renal disease; ERT: Enzyme replacement therapy; ASO: Antistreptolysin O; MRI: Magnetic resonance imaging; HRM: High resolution melting.

## Competing interests

The authors declare that they have no competing interests.

## Authors’ contributions

FI performed clinical analyses and drafted the manuscript; FP conducted the experiments and drafted the manuscript; GA carried out the enzymatic analyses; CZ participated in the experiments and in the drafting of the manuscript; PC performed the genetic analysis and participated in the drafting of the manuscript; SS participated in the experiments; CB participated in the experiments; GD planned and coordinated the study, including obtaining the informed consent and the ethic approval to conduct the study. All authors read and approved the final manuscript.
